# Linking periodontal disease with obesity and blood glucose

**DOI:** 10.6026/97320630017691

**Published:** 2021-07-31

**Authors:** Ena Sharma, Deepak Sharma, Amit Lakhani, Ankit Mahajan, Rasveen Kaur

**Affiliations:** 1 Maharishi Markandeshwar College of Dental Sciences and Research, Mullana, Ambala Haryana, India; 2 HP Government Dental College, Himachal Pradesh, India; 3 Dr BR Ambedkar State Institute of Medical Sciences, India

**Keywords:** obesity, blood glucose, periodontal

## Abstract

It is of interest to evaluate the association of obesity and blood glucose level with periodontitis. Patients (150 with age range 26-68 years) were included based on WHO obesity criteria, undiagnosed for periodontitis, with Body Mass Index (BMI) ≥ 30 and systemically healthy. These patients underwent periodontal examination followed by blood analysis for lipid profile and blood sugar level. The periodontal status was determined using parameters such as Plaque index (PI), gingival index (GI), Probing depth (PPD) and Clinical attachment loss (CAL). 103 (68.7%) patients had >190 of triglyceride values. Data shows that periodontitis has no statistical significance with total cholesterol, HDL, LDL and moderate significance with VLDL, triglycerides. Glycemic control of the patients is assessed using postprandial blood sugar (PPBS) and Fasting Blood Sugar (FBS). Data shows that 129(86.6%) had FBS (mg/dl) <100 and 21 (14.0%) had FBS (mg/dl) >100. So the number of patients with FBS (mg/dl) < 100 were more i.e., 129 (86.6%). The PPBS values were in 136 (90.7%) had PPBS (mg/dl) <140 and only 14(9.3%) had PPBS (mg/dl) >140 group of patients were said have glucose intolerance. Thus, there is no change in lipid profile with established periodontitis in obese individuals. However, altered glycemic control is observed.

## Background:

Obesity (BMI > 30) has reached 5% of the country's population. [1] Being overweight and obese are important risk factors for various adult diseases, including type-2 diabetes, hyperlipemia, cholelithiasis, cardiovascular and cerebrovascular disease like hypertension and arteriosclerosis. [2] Type 2 diabetes is prevalent in India. [3 - check with author] Obesity has emerged as one of the risk indicators of periodontal disease. Periodontitis is characterized by chronic inflammation of the supporting structures of the teeth. [4] The etiologic agents of this disease are mainly gram-negative bacteria existing in a complex biofilm in the sub gingival region. Lipopolysaccharides (LPS) and other virulence factors of these bacteria have been shown to promote a host-mediated, tissue destructive immune response that leads to gingival inflammation, destruction of periodontal tissue, loss of alveolar bone, and eventual exfoliation of teeth. [4].Obesity and excessive dietary fat intake can lead to prolonged hyperlipidaemia (HL) [5,6] which is known to have a profound effect on the function and activation state of myeloid cells. [7] A short-term high fat diet results in prolonged impairment in the antibacterial function of polymorphonuclear leukocytes (PMNs), and an increased release of superoxide anions in response to cell agonists (PMN "priming"). Activated or primed PMNs have been linked to damage of the periodontal tissues. [8] Several studies have indicated that severe periodontitis is associated with a modest decrease in high density lipoprotein (HDL) cholesterol, a modest increase in low density lipoprotein (LDL) cholesterol, and a more robust increase in plasma triglycerides (TG). [9,10, 11,12,13] These biological signalling molecules from local inflammation into the circulation have myriad physiological effects promoting enhanced lipogenesis, increased lypolisis and reduced lipid clearance. The end result is hyperlipidemia or an accumulation of serum free fatty acids (FFA), LDL, and TG. On the other hand, a possible role of hyperlipidemia for periodontitis is also obvious from several studies. Hyperlipidemia is known to cause a hyperactivity of white blood cells [13,14] Hyperactivity of white cells, e.g., increased production of oxygen radicals has been shown to be frequently associated with progressive periodontitis in adults. [15-17]. Therefore, it is of interest to relate periodontal diseases with obesity and blood glucose (Figure 1 - see PDF).

## Methodology:

### Data source:

This study included 150 patients visiting the Department of Periodontics, Bangalore, India.

### Inclusion criteria:

Both male and female patients aged 20 years and above were included in the study.

Patients having BMI ≥ 30

Systemically healthy subjects.

### Exclusion criteria:

Patients who are already under treatment for obesity.

Known diabetic patients

Patients who are smokers

Patients with history of any antibiotics or analgesics therapy for 3 months prior to the study enrollment.

Patients who underwent periodontal therapy for last six months.

### Data collection:

Patients coming to the OPD of Department of Periodontics were screened for their Body Mass Index (BMI). The nature and purpose of the study was explained to the patients and written consent was obtained. A detailed systemic case history was taken with gingival index (Loe and Silness, 1963), Plaque index (Glickman Modification of the Quigley and Hein). Plaque Index (1970), Probing depth (PD), Clinical attachment loss (CAL) followed by BMI measurement. BMI was calculated my measuring height of the patient in meter and weight of the patient in Kilogram. BMI was calculated using WHO formula (Kg/m^2^). 150 patients BMI was > 30 according to the WHO chart were included in the study. The selected patients were subjected to blood investigation for their lipid profile and blood glucose level. Serum levels of triglycerides, High Density lipoprotein (HDL) Cholesterol, Low Density lipoprotein (LDL) Cholesterol, Very Low Density lipoprotein (VLDL) Cholesterol and Total Cholesterol were analyzed using enzyme assays. Fasting Blood glucose level and Post prandial Blood glucose level was assessed.

## Results:

**Figure 2:**
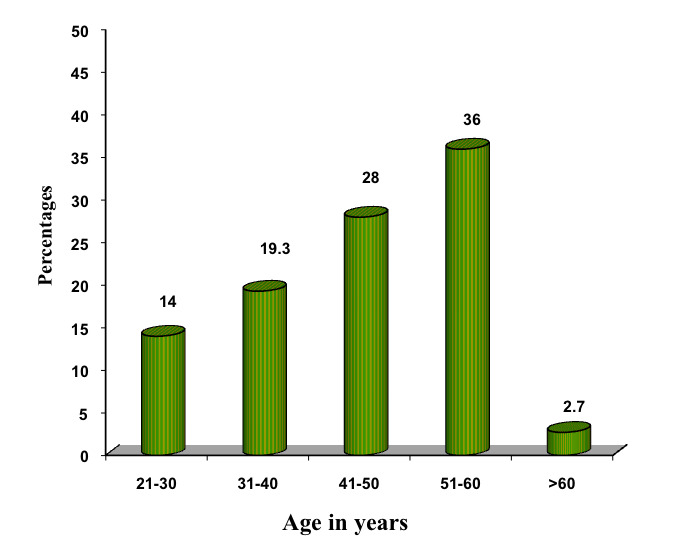
Age distribution of patients

**Figure 6:**
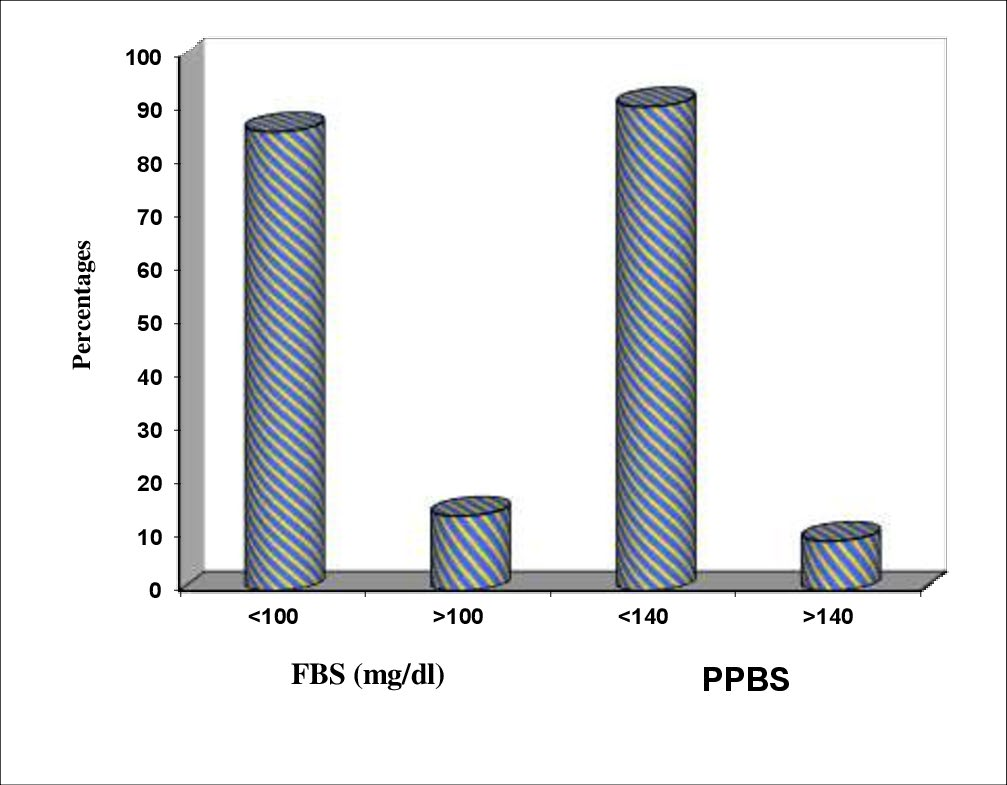
Blood sugar parameters

An Observational Co-relational study consisting of 150 patients with BMI ≥ 30 whose clinical parameters like PI, GI, PPD and CAL are compared to lipid profile and blood glucose level to find out the association of obesity with blood glucose level and to evaluate the association of obesity and blood glucose level with periodontitis. Clinical parameters of all the patients with ≥ 30 BMI were evaluated and are shown in Table 1(see PDF) and [Figure 2] to [Figure 6].

## Comparison of PI, GI, PPD and CAL with Total cholesterol

**Figure 7:**
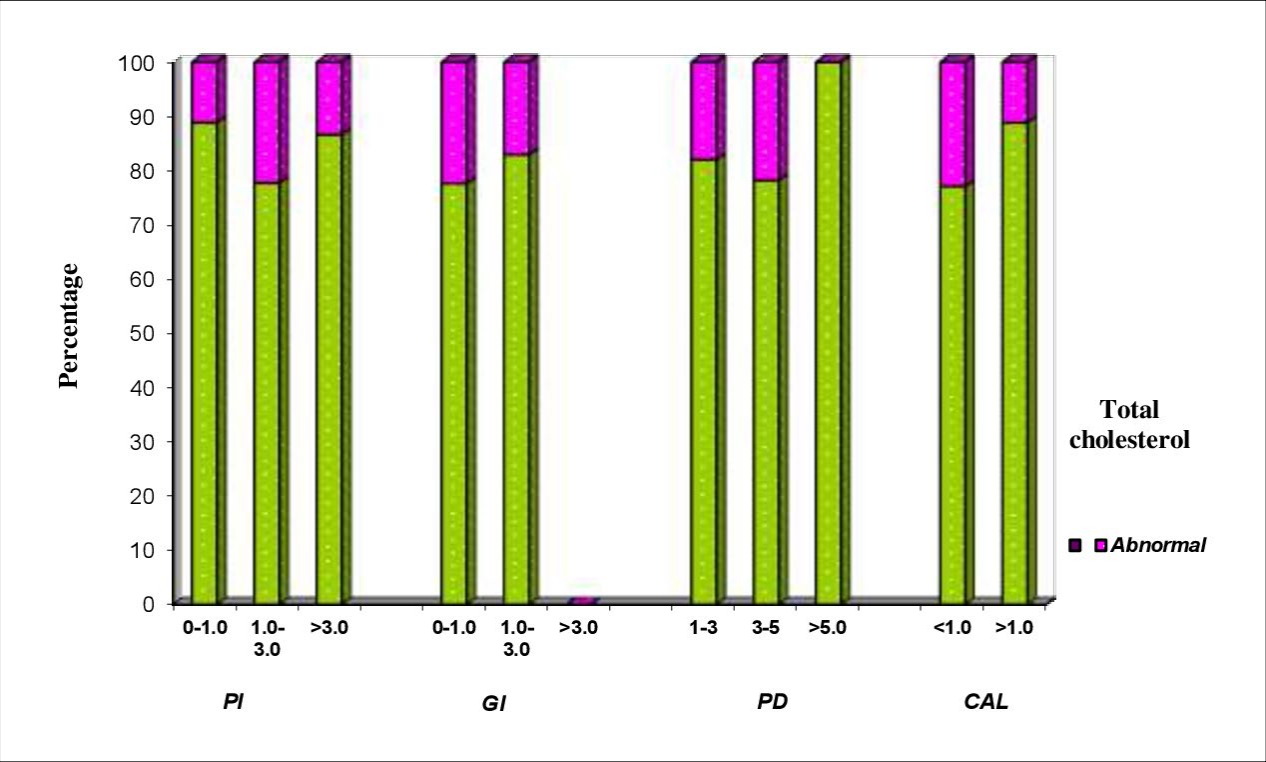
Correlation of clinical parameters of patient’s and total cholesterol

Clinical parameters of 150 patients are compared with normal and abnormal total cholesterol levels (Table 2 - see PDF,Figure 7). Total cholesterol level of <200(mg/dl) is considered as normal and >200(mg/dl) is considered as abnormal. Abnormal total cholesterol level ranged from 11.1% to 22.2 % in all the three PI indices, 17 % to 22.3% in all the three GI indices, 17.9% to 21.7% in all the four PPD scores, 11% to 22.8 % in both CAL scores and P value showed no statistical significance in any score.

## Comparison of PI, GI, PPD and CAL with HDL

Clinical parameters of 150 patients are compared with normal and abnormal HDL levels (Table 3 - see PDF). HDL level of <35(mg/dl) is considered as normal and >35(mg/dl) is considered as abnormal. Abnormal HDL level ranged from 69.8% to 80 % in all the three PI indices, 66% to 74.8% in all the three GI indices, 50% to 75.8 % in all the four PPD scores and P value showed no statistical significance in any score.

## Comparison of PI, GI, PPD and CAL with LDL

Clinical parameters of 150 patients are compared with normal and abnormal LDL levels (Table 4 - see PDF). LDL level of <120(mg/dl) is considered as normal and >120(mg/dl) is considered as abnormal. Abnormal LDL level ranged from 13.3 % to 33.3 % in all the three PI indices 23.4% to 28.2% in all the three GI indices, 25% to 27.5 % in all the four PPD scores, 14.8 % to 29.3 % in both CAL scores and P value showed no statistical significance in any score.

## Comparison of PI, GI, PPD and CAL with VLDL

Clinical parameters of 150 patients are compared with normal and abnormal VLDL levels (Table 5 - see PDF). VLDL level of <35(mg/dl) is considered as normal and >35(mg/dl) is considered as abnormal. Abnormal VLDL levels showed no statistical significant difference in all the three PI and GI indices, in four PPD and in two CAL scores.

## Comparison of PI, GI, PPD and CAL with Triglycerides

**Figure 8:**
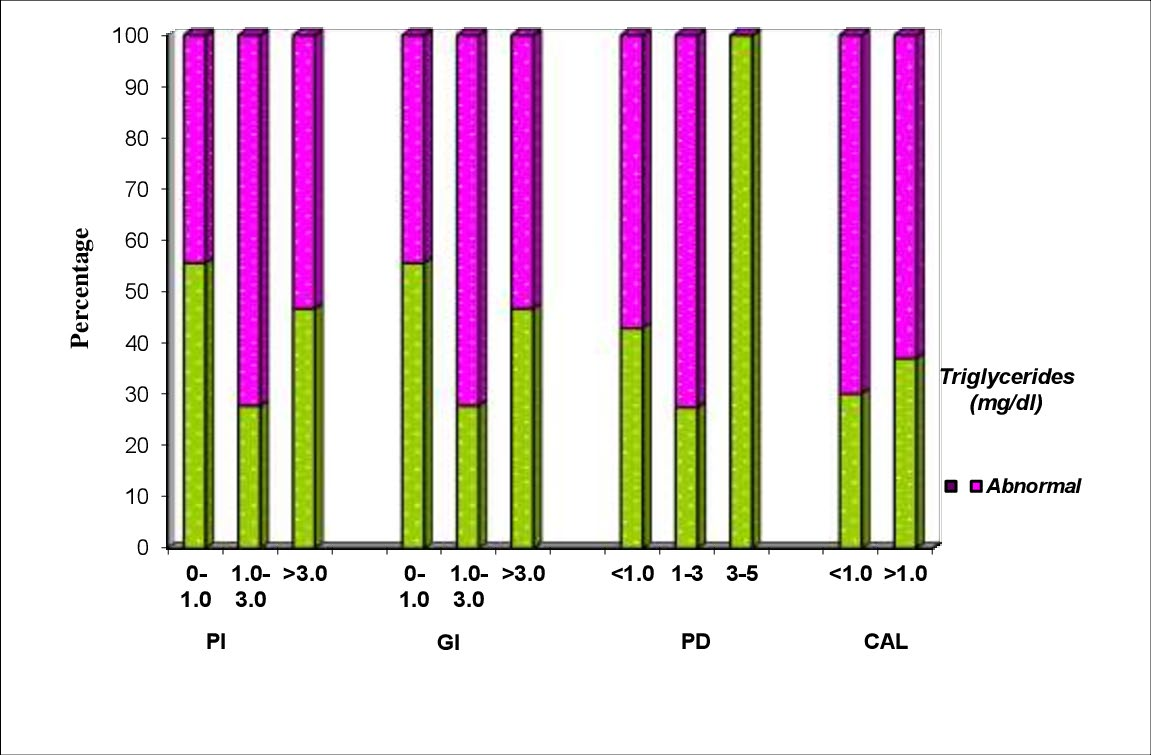
Correlation of clinical parameters of patients and triglycerides

Clinical parameters of 150 patients are compared with normal and abnormal Triglycerides levels (Table 6 - see PDF & Figure 8). Triglycerides level of <190(mg/dl) is considered as normal and >190(mg/dl) is considered as abnormal Abnormal Triglycerides level ranged from 44.4% to 72.2% in all the three PI indices, 64.1% to 78.7 % in all the three GI indices, 57.1% to 72.5 % in all the four PPD scores, 63% to 69.9% in both CAL scores and P value showed no statistical significance in any score.

## Comparison of PI, GI, PPD and CAL with FBS

**Figure 9:**
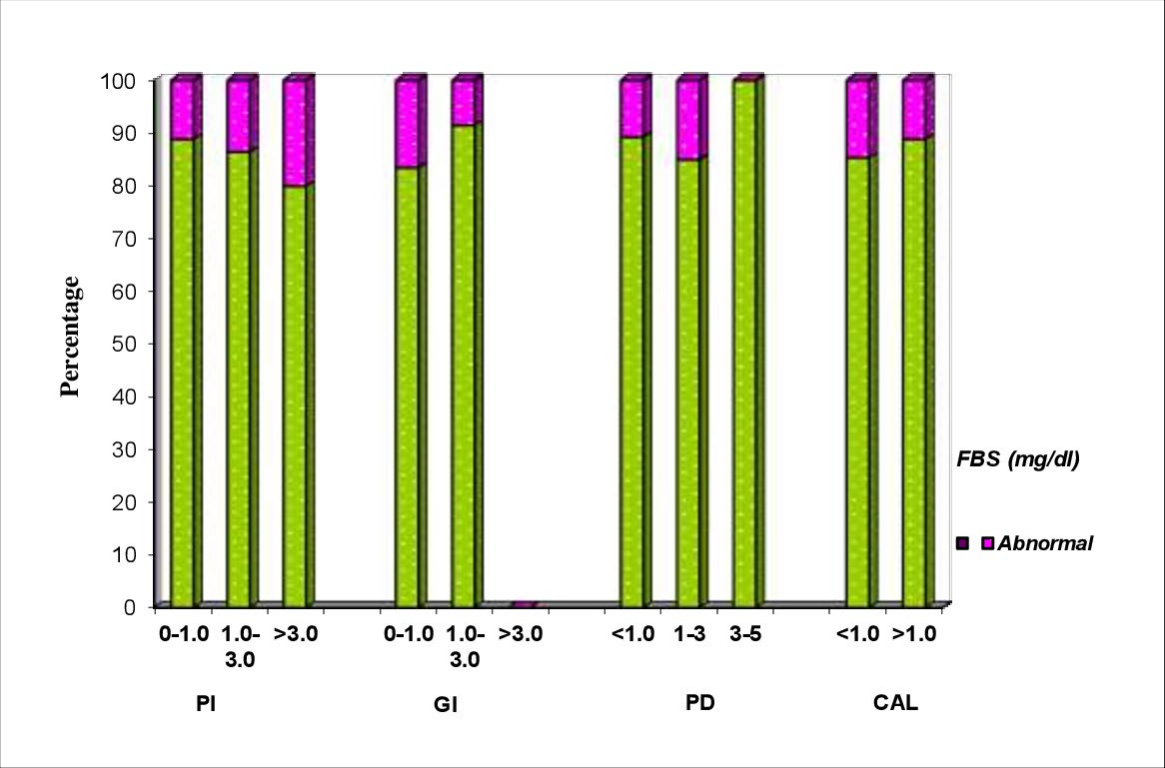
Correlation of clinical parameters of patients and FBS

Clinical parameters of 150 patients are compared with normal and abnormal FBS (Table 7 - see PDF, Figure 9). FBS of <100(mg/dl) is considered as normal and >100(mg/dl) is considered as abnormal. Abnormal FBS ranged from 11.1% to 20% and 8.5% to 16.5% in all the three PI and GI indices, respectively and P value showed no statistical significance. Abnormal FBS ranged from 15% to 10.7% in all the four PPD scores and P value showed statistical significance.

## Comparison of PI, GI, PPD and CAL with PPBS

**Figure 10:**
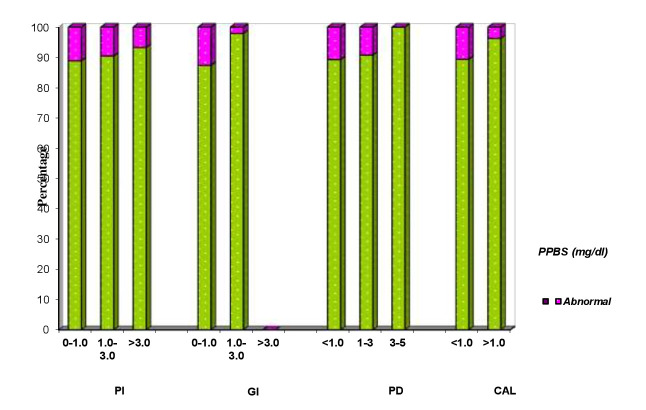
Correlation of clinical parameters of patients and PPBS

Clinical parameters of 150 patients are compared with normal and abnormal PPBS (Table 8 - see PDF, Figure 10). PPBS of <140 (mg/dl) is considered as normal and >140(mg/dl) is considered as abnormal. Abnormal PPBS ranged from 6.7% to 11.1% in all the three PI indices and P value showed no statistical significance. Abnormal PPBS ranged from 2.1% to 12.6% in all the three GI indices and P value showed statistical significance. Abnormal PPBS showed no statistical significant differences in all the four PPD scores and in both CAL scores.

## Discussion:

Increased levels of obesity have been shown to result in a decline in life expectancy. [14] It is well known that there is a causal relationship between serum lipid levels and systemic health, particularly cardiovascular disease, diabetes, tissue repair capacity, immune cell function, and serum levels of pro-inflammatory cytokines. On the other hand, periodontitis induced changes in immune cell function may cause metabolic dysregulation of lipid metabolism through a mechanism involving pro-inflammatory cytokine. [15] It was demonstrated that in subjects with hyper-cholestrolaemia and cardiovascular disease had a significantly worse periodontal condition than control subjects. Katz et al. conducted a study on the association between hyper cholesterolaemia, cardiovascular disease, and severe periodontal disease and found a significant association of patients with hyper cholesteroalaemia and CPITN scores of IV. [16] Many studies showed association and many hypotheses were also put forward but needed clinical studies in multi centre to find out the same. The BMI measured is categorized using the World Health Organization (2000) classification. [17] Patients were included in our study based on BMI and not based on known cases of periodontitis or systemic diseases. BMI is used as an indicator of obesity. The obese patients who had BMI of over 30 kg/m2 were included in the study and that followed periodontal examination and blood investigations. Majority of our patients were adults and older age group than young adults. Hyper lipidemia is a causative factor for cardiovascular diseases and can contributes to periodontitis. In our study the levels of plasma lipids in these obese patients were compared with clinical periodontal findings in order to evaluate the association between hyper lipidemia and periodontitis.

Clinical parameters to assess periodontal status included Plaque index, gingival index, Probing depth and Clinical attachment loss. 84.0% of the patients had PI index of 1.0-3.0 and 10% had >3.0. 68.7% of patients had GI index of 0-1.0 and 31.3% had 1.0-3.0. The indices of PD and CAL, which are the indicators of the established periodontitis, were as follows. We found that 80.0% of study patients had PPD of 1-3 and 18.7% had <1.0. In CAL 82.0% had indices of <1.0 and 18.0% had >1.0. This was indicative of our finding that majority of our study patients had no established periodontitis. The results in our study are indicative of majority of the patients had BMI of over 30 kg/m2 no higher values of lipid profiles. As the study was entirely in not known cases of periodontitis, the results are indicative of fact that lipid profiles are not higher in increased BMI patients. This can also indicative of that established periodontitis in increased BMI patients could have higher lipid values as postulated earlier. Hyper lipidaemia is a state of abnormal lipid profile, and is suggested that it could be associated with periodontitis, although the role of hyper lipidaemia as a risk factor has not been established. [18] The association between altered lipid profile and periodontitis has been investigated in several studies. [19,20] Hyperlipidemia has been suggested to be one possible mechanism explaining the association between obesity and periodontitis, an association, which has been found in several cross-sectional studies in recent years. [21,22].

We found no significant association of periodontitis with the lipid profiles like Total cholesterol, HDL, LDL and VLDL except for Triglycerides, which showed moderate significance. Number of patients with periodontitis in our study was too less to have a statistical analysis to compare abnormal values in the lipid profiles as well as blood sugar values. But with the close observation from the comparison tables of lipid profile values to CAL indices >1.0 shows that abnormal values of Total cholesterol, HDL, LDL, VLDL and Triglycerides are 3(11.1%), 8(29.6%), 4(14.8%) and 17(25.9%) respectively. This indicates that there is no significant association of periodontitis with the abnormal lipid values except triglycerides, which showed abnormal value in 25.9% of patients with periodontitis. In the present study diabetic subjects were excluded because of the complex association between diabetes, dyslipidaemia and periodontitis, which may prevent accurate controlling. FBS and PPBS were obtained in all the patients who were not known diabetics. The results of our study are, out of 150 patients in the study, 129(86.6%) had FBS (mg/dl) <100 and 21 (14.0%) had FBS (mg/dl) >100. So the number of patients with FBS (mg/dl) <100 were more i.e., 129(86.6%). When observations are made for PPBS in 150 patients, 136(90.7%) had PPBS (mg/dl) <140 and 14(9.3%) had PPBS (mg/dl) >140. So the number of patients with PPBS (mg/dl) <140 were more i.e., 136(90.7%). Hence our study showed that most of the patients had good glycemic control and only 14.0% had increased FBS and 9.3% had increased PPBS. These can be the group of patients with impaired glucose tolerance. Examination of the available data reveals strong evidence that diabetes is a risk factor for gingivitis and periodontitis, and the level of glycemic control appears to be an important determinant in this relationship. This can be explained in our study that our patients did not show abnormal blood sugar level because neither they were diagnosed diabetics nor periodontitis. So it can be concluded that if diabetes and periodontists coexist, then this can alter the glycemic control of the patients.

Metabolic diseases and their link to risk indicators of periodontitis are known. [26] Their study sample consisted of one hundred patients classified as having impaired glucose tolerance but no manifest diabetes, hyperlipademia, or abnormal metabolic status. The findings suggested that abnormal glucose tolerance, which is a predisposing factor for diabetes mellitus, does not appear to be a risk indicator for periodontal disease. Our study is also of the similar conclusion that smaller group of patients that is 14.0% of increased FBS and 9.3% of increased PPBS had impaired glucose tolerance, did not show significant correlation with the periodontitis. Recent studies have reported a relationship between obesity and periodontal disease. So obesity is the strongest risk factor for type-2 diabetes, which is a risk factor for periodontal disease. An oral glucose tolerance test is necessary to diagnose diabetes in these impaired glucose tolerance group of patients. Saito et al. in their study found that impaired glucose tolerance seemed to have no association with either deep pockets or severe attachment loss in any multivariate model, despite the greater number of subjects (n ¼ 108), as compared with diabetes (n ¼ 40). Impaired glucose tolerance, which is an intermediate glucose condition between diabetes and normal glucose tolerance, may not have any effect on periodontal disease. This concur with our report, in which periodontal clinical parameters did not show significant correlation with blood sugar levels but can lead to impaired glucose tolerance. [27].

Hyperlipidaemia and hyperglycaemia are major risk factors for cardiovascular disease. In recent years, some evidence has been presented that periodontal disease is associated with an increased risk of cardiovascular disease. To further elucidate this association, Lo¨sche et al. studied standard blood chemistry variables known as risk markers for cardiovascular disease in periodontally diseased and healthy subjects.28 Total cholesterol, low density lipoprotein cholesterol and triglycerides were significantly higher in periodontally diseased subjects by about 8% (p, 0.03), 13% (p, 0.003) and 39% (p, 0.001), respectively, when compared to controls. Although subjects with diabetes were excluded from the study, they found significantly higher blood glucose levels in the patient than in the control group (85°25 versus 73°17 mg/dl; p, 0.02). There was also a significantly higher frequency of pathological plasma lipid profiles in the patient than in the control group. The results indicate that hyper lipaemia and pre-diabetes may be associated with periodontal disease in systemically healthy subjects. These data do not allow us to decide, whether periodontal disease causes an increase in hyperlipidaemia and in a prediabetic state or whether periodontal disease and cardiovascular disease share hyperlipidaemia and the prediabetic state as common risk factors. [28] If obesity is a true risk factor for periodontal disease, the association among periodontal disease, obesity, and type 2 diabetes or cardiovascular disease must be very complex because each is a confounding factor for the other. In addition, several studies have suggested that periodontal disease affects both glucose and lipid metabolism. These are important factors in the development of both type 2 diabetes and cardiovascular disease. Thus, we must point out that our results differ from most previous studies, which have mainly shown a fairly strong association between periodontal infection and unfavourable lipid composition.

## Conclusion:

Analysis of epidemiology data shows that periodontal diseases are not signficantly linked with obesity. There is no change in lipid profile with established periodontitis in obese individuals. However, altered glycemic control is observed.

## Limitation:

A prospective cohort study with different age groups and sexes is required in this context.

## Figures and Tables

**Figure 3:**
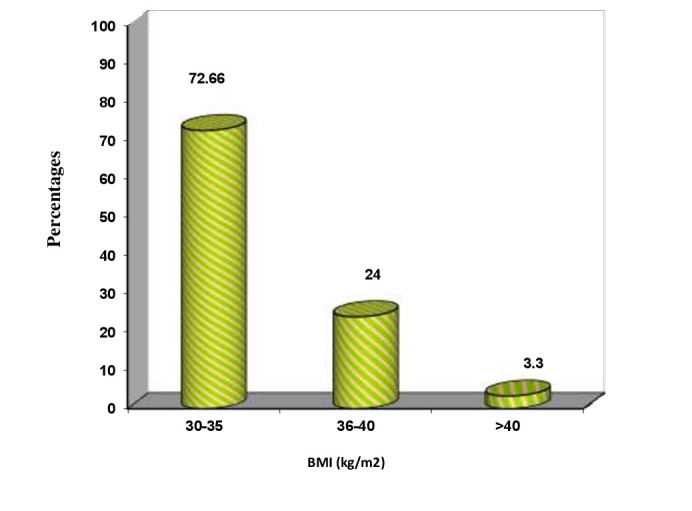
BMI distribution

**Figure 4:**
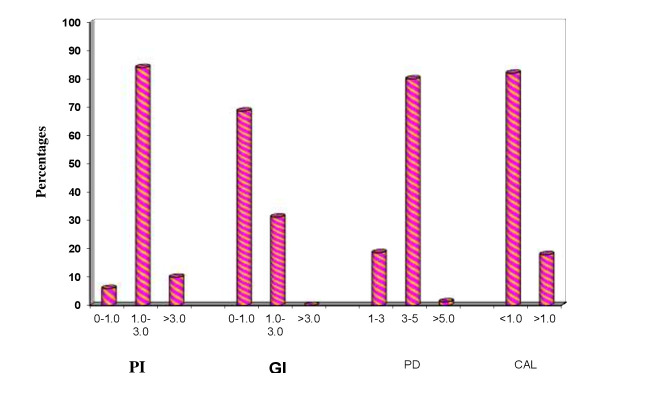
Clinical parameters

**Figure 5:**
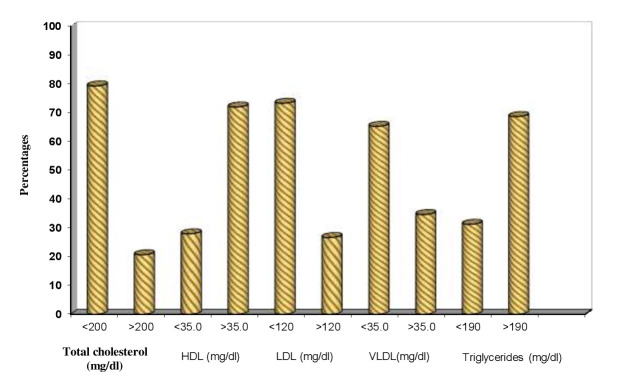
Lipid parameters
